# Reversible Actuation Ability upon Light Stimulation of the Smart Systems with Controllably Grafted Graphene Oxide with Poly (Glycidyl Methacrylate) and PDMS Elastomer: Effect of Compatibility and Graphene Oxide Reduction on the Photo-Actuation Performance

**DOI:** 10.3390/polym10080832

**Published:** 2018-07-28

**Authors:** Josef Osicka, Miroslav Mrlik, Marketa Ilcikova, Barbora Hanulikova, Pavel Urbanek, Michal Sedlacik, Jaroslav Mosnacek

**Affiliations:** 1 Centre of Polymer Systems, University Institute, Tomas Bata University in Zlín, Trida T. Bati 5678, 760 01 Zlín, Czech Republic; osicka@utb.cz (J.O.); mrlik@utb.cz (M.M.); hanulikova@utb.cz (B.H.); urbanek@utb.cz (P.U.); 2 Polymer Institute, Slovak Academy of Sciences, Dúbravska cesta 9, 845 41 Bratislava, Slovakia; upolmosj@savba.sk; 3 Centre for Advanced Materials Application, Slovak Academy of Sciences, Dúbravska cesta 9, 845 11 Bratislava, Slovakia

**Keywords:** graphene oxide, reduction, SI-ATRP, photo-responsive material, light-stimuli material, dielectrics, poly (glycidyl methacrylate), dynamic mechanical analysis

## Abstract

This study is focused on the controllable reduction of the graphene oxide (GO) during the surface-initiated atom transfer radical polymerization technique of glycidyl methacrylate (GMA). The successful modification was confirmed using TGA-FTIR analysis and TEM microscopy observation of the polymer shell. The simultaneous reduction of the GO particles was confirmed indirectly via TGA and directly via Raman spectroscopy and electrical conductivity investigations. Enhanced compatibility of the GO-PGMA particles with a polydimethylsiloxane (PDMS) elastomeric matrix was proven using contact angle measurements. Prepared composites were further investigated through the dielectric spectroscopy to provide information about the polymer chain mobility through the activation energy. Dynamic mechanical properties investigation showed an excellent mechanical response on the dynamic stimulation at a broad temperature range. Thermal conductivity evaluation also confirmed the further photo-actuation capability properties at light stimulation of various intensities and proved that composite material consisting of GO-PGMA particles provide systems with a significantly enhanced capability in comparison with neat GO as well as neat PDMS matrix.

## 1. Introduction

Smart materials belong to a group of matters. Such physical properties can be changed upon external stimulus i.e., electric [1,2,3,4] or magnetic field [5,6,7,8], pH [9,10,11,12], temperature [13,14,15,16], or light [17,18,19,20]. The systems exhibiting reversible photo-actuation and light-responsive properties are vital for the research community only during the last decade while the rest of the above mentioned properties have been part of the research community over the past 30 years. Therefore, there are still many drawbacks in this topic like stability of the systems or even performance, which reversibly contracts or elongates the polymer composite sample upon light stimulation. Materials exhibiting this phenomenon can find utilization in various systems controlling the damping properties [21], sensing [22], or haptic displays [23]. Therefore, the broad applications belong to the field of electronic [24], civil engineering [25], or medicine [26], respectively.

In these applications, the graphene oxide (GO) particles as a part of smart systems were properly investigated due to their unique properties, i.e., good dispersibility in the surrounding media [27], possible post-functionalization [28], are easily reduced, and, therefore, provide tunability of the electrical conductivity [29]. There are various approaches regarding how to reduce the GO particles such as treatment with hydrazine [30], reduction in the acidic environment [31], or simultaneous reduction of GO during the surface-initiated atom transfer radical polymerization (SI-ATRP) [32].

Due to the high requirements in the above mentioned applications on the final material and from the mechanical and stability point of view, the utilization of poly (dimethyl siloxanes) PDMS is very frequent [33]. Such PDMS material can be either tailored by cross-linking density or using the addition of silicone oil and a curing agent. However, there is still a lack of compatibility of PDMS with common fillers such as MWCNT, graphene, or even GO and, therefore, such fillers need to be modified in order to improve compatibility and dispersibility as well as overall properties of prepared composite materials. There are various techniques of modification using surfactants [34], but such systems are rather instable or uncontrolled polymerization from the surface, which significantly changes the properties of filler [35] and, therefore, the SI-ATRP approach seems to be a very convenient method of surface modification using a variety of monomers [27,32,36,37] or copolymers [38], which significantly improves the compatibility while the basic physical properties such as mechanical or electrical are affected only negligibly.

Therefore, this article is focused on the SI-ATRP of glycidyl methacrylate (GMA) from the surface of GO particles in order to improve their compatibility with PDMS. Successful modification of GO by short polymer chains was proved using TEM and TGA-FTIR measurements. The compatibility, mechanical, dielectric, and photo-actuation properties were investigated when special aim was concentrated on the effect of light intensity on the final photo-actuation performance.

## 2. Materials and Methods

Graphite (powder, <20 μm, synthetic), sulfuric acid (H_2_SO_4_, 95%–98%), sodium nitrate (NaNO_3_, ≥99%), potassium permanganate (KMnO_4_, 97%), hydrogen peroxide (H_2_O_2_, 29.0–32.0 wt %), α-bromoisobutyryl bromide (BiBB, 98%), triethylamine (TEA, ≥99%), glycidyl methacrylate (GMA, 98%), ethyl α-bromoisobutyrate (EBiB, 98%), *N*,*N*,*N*′,*N*″,*N*″-pentamethyldiethylenetriamine (PMDETA, ≥99%), copper bromide (CuBr, ≥99%), anisole (99%), and diethyl ether (≥99%) were all purchased from Sigma Aldrich and were used as received.

The graphene oxide was fabricated from graphite powder by a modified Hummers method [39]. The product was separated in a high-speed centrifuge (Sorvall LYNX 4000, Thermo Scientific, Waltham, MA, USA) operating at 10,000 rpm for 20 min at 25 °C. The cleaning routine was based on the dispersion of the GO in 0.1 M HCl and their re–separation in a centrifugal field. The procedure was repeated with distilled water several times until the pH has reached a value of 7. Afterward, the particles were lyophilized in order to remove the residual amount of water after purification and remove the brown powder that was obtained. The initiator BiBB was immobilized, according to the procedure described in detail elsewhere [32]. Two various polymerizations from the surface of GO particles in order to prepare poly (glycidyl methacrylate)-modified GO (GO-PGMA and GO-PGMA-2), were performed by the following procedure. The molar ratio of reactants [GMA]:[EBiB]:[CuBr]:[PMDETA] was [100]:[1]:[1]:[4] and [100]:[1]:[1]:[2] while anisole (50 vol. %) was used as a solvent. The presence of oxygen was minimized by degassing the system by several freeze-pump-thaw cycles and after the last cycle by filling the system with argon. Lastly, the CuBr catalyst was added under argon flow and polymerization was carried out at 60 °C for 4 h and at 50 °C for 12 h for polymerizations performed with 4:1 and 2:1 ratio of PMDETA to CuBr, respectively. The product was purified by filtration using DMF, acetone, and diethyl ether and dried using lyophilization.

**Figure 1:**
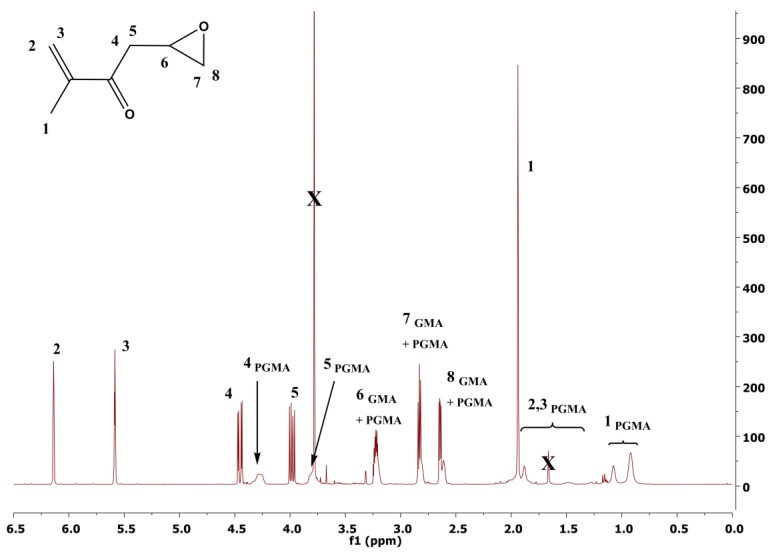
Representative ^1^H NMR spectrum from filtered polymerization mixture of glycidyl methacrylate performed with [GMA]:[EBiB]:[CuBr]:[PMDETA] ratio of [100]:[1]:[1]:[4] at 60 °C for 4 h. The monomer conversion was 43%.

**Figure 2:**
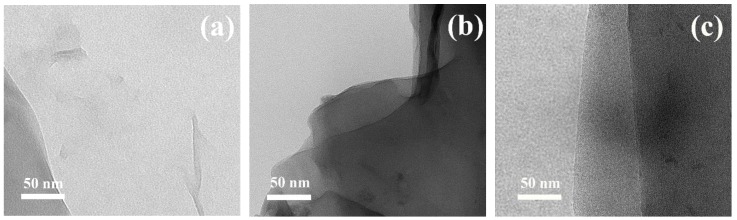
TEM images of (**a**) neat GO; (**b**) GO-PGMA; and (**c**) GO-PGMA-2.

^1^H nuclear magnetic resonance (NMR) spectra were recorded at 25 °C using an instrument (400 MHz VNMRS Varian, Tokyo, Japan) with deuterated chloroform (CDCl_3_) as a solvent. The ^1^H NMR was used to determine the monomer conversion from the ratio of area of peaks assigned to PGMA to the area of peaks assigned to both PGMA and GMA (Figure 1). The molar mass and polydispersity (*Ð*) of PGMA chains were investigated using gel permeation chromatography (GPC) on the GPC instrument (PLGPC220, Agilent, Hachioji, Japan) equipped with GPC columns (Waters 515 pump, two PPS SDV 5 lm columns (diameter of 8 mm, length of 300 mm, 500 Å + 105 Å)) and a Waters 410 differential refractive index detector tempered to 30 °C. The neat GO and GO with a grafted PGMA polymer layer were observed using a transmission electron microscope (TEM, JEM-2100Plus, Jeol, Tokyo, Japan). The samples for the TEM analysis were prepared by dispersing the particles in acetone using mechanical stirring for 5 and 2 min of sonication and dropping the resultant suspension onto a copper grid. The Raman Shift (3 scans, resolution of 2 cm^−1^) were collected on a Nicolet DXR (Nicolet, Rhinelander, WI, USA) using an excitation wavelength of 532 nm. The integration time was 30 s while the laser power on the surface was set to 1 mW. The powders under investigation were compressed to the form of pellets (diameter of 13 mm, thickness app 1 mm). The pellets were used for electrical conductivity measurements using a two-point method (Keithley 6517B, Cleveland, OH, USA). The contact angle (CA) values were evaluated from the static sessile drop method on the pellets carried out on a Surface Energy Evaluation system equipped with a CCD camera (Advex Instruments, Brno, Czech Republic). A droplet (5 μL) of PDMS was carefully dripped onto the surface and the CA value was recorded. The presented CA results are the average values from 10 independent measurements. The thermo-oxidation decomposition of the samples was on-line monitored using a thermogravimetric analyzer (TGA) operating in an oxygen atmosphere coupled with FTIR with a help of Nicolet iS10 equipped with TGA-IR module (Thermo Scientific, Waltham, MA, USA). In the Figure 2, the highlighted bands are those where the FTIR signal was collected in order to prove the presence of the various components in the GO-based powders.

The composites containing various amount of GO-based particles were prepared using the following standard procedure: PDMS (Sylgard 184, Atlanta, GA, USA) was mixed with silicone oil and subsequently a cross-linker was added in ratios of PDMS: silicone oil: cross-linker 7/3/1 and, lastly, 0.1 vol. % of the particles was added before the curing. In the case of the neat PDMS matrix, we further investigated the same ratio of PDMS in this paper. Silicone oil and a cross-linker was used but without the addition of the particles. In the case of all samples, the mixtures were degassed using a vacuum oven in the four cycles at 10 mbar at room temperature and then placed into the oven and cured at an elevated temperature set to 60 °C for 6 h.

The thermal conductivity was measured by one side contact method using the TCi model (C-Term Technologies, Vancouver, BC, Canada). The viscoelastic properties of both the nanocomposite and pure polymer matrix were studied through the dynamic mechanical analysis (DMA) in tensile mode. All measurements were performed at a linear viscoelastic region. The measurement was done at 1 Hz in the temperature range from −150 to 150 °C. To provide information about how the modification of the GO with PGMA grafts influences the strain of the materials, a dependence of the storage and loss moduli was plotted against the change in the length Δ*L*, i.e., parameter specified in the experimental photo-actuation investigations. The measurements were performed at 1 Hz and at 25 °C. The dielectric spectroscopy ranged from a temperature of −150 to 100 °C and ranged in frequency from 10^−1^ to 10^7^ Hz, which were employed to investigate the polymer chains dynamics.

The glass transition process was evaluated through activation energies calculated from the Arrhenius equation (Equation (1)) in order to see the effect of modification on the relaxation processes in the PDMS based composites.

(1) $$f_{\beta }=f_{\infty }\exp\left(\frac{E_{a}}{k_{B}T}\right),$$

where *E*_a_ is the activation energy, $f_{\infty }$ is the pre-exponential factor, *T* is thermodynamic temperature, and *k_B_* is the Boltzmann constant.

In order to properly investigate the polymer chains dynamics, the loss permittivity need to be recalculated to the loss modulus. This recalculation was based according to Equation (2) [40].

(2) $$\begin{array}{l} M^{*}=\frac{1}{\varepsilon^{*}}\\ M^{\prime }=\frac{\varepsilon^{\prime }}{{\varepsilon^{\prime }}^{2}+{\varepsilon^{\prime \prime }}^{2}}\\ M^{\prime \prime }=\frac{\varepsilon^{\prime \prime }}{{\varepsilon^{\prime }}^{2}+{\varepsilon^{\prime \prime }}^{2}} \end{array}$$

where ε* is complex permittivity and *ε*′ and *ε*″ are relative permittivity and loss permittivity, respectively. M* is the complex dielectric modulus and *M*′ and *M*″ are storage and dielectric loss moduli, respectively.

The photo-actuation ability of both the matrix and composite samples was investigated using thermal mechanical analysis (TMA, Mettler Toledo, Columbus, OH, USA), which was previously published [15]. Red LED diode (Luxeon Rebell, Philips, Amsterdam, the Netherlands) was used for irradiation. Irradiation was applied for 10 s at 627 nm with 6, 9, and 12 mW light source intensity under 10% pre-strain of the samples. The maximum value of actuation is characterized by a change in sample length during the exposition to light, Δ*L* = (*L*_0_ − *L*)/*L*_0_, where *L*_0_ is the length of non-irradiated sample and *L* is the length of an irradiated sample.

## 3. Results and Discussion

Successful polymerization of GMA from the surface of GO was confirmed by various techniques such as ^1^H NMR, GPC, TEM, and TGA-FTIR. The conversion of GMA was calculated to be 43% and 46%, according to ^1^H NMR spectra and the molar mass and Ð of PGMA chains determined from GPC were 5900 g·mol^−1^ and 1.28 and 6100 g·mol^−1^ and 1.26 for the GO-PGMA 1 and GO-PGMA 2, respectively, fit the experimental molar mass with the theoretical one. Furthermore, from the TEM investigation, the 2D nature of the neat GO particles (Figure 2a) can be clearly seen. Moreover, the 2D structure was also observed in the case of GO-PGMA while the presence of the PGMA chains exhibit rather flossy-like structures, which provided substantial coating for the GO particle (Figure 2b). The change in the reaction mixture for GO modification (Figure 2c) does not lead to a significant change in the morphology and provides nearly the same morphology than the previous one (Figure 2b).

**Figure 3:**
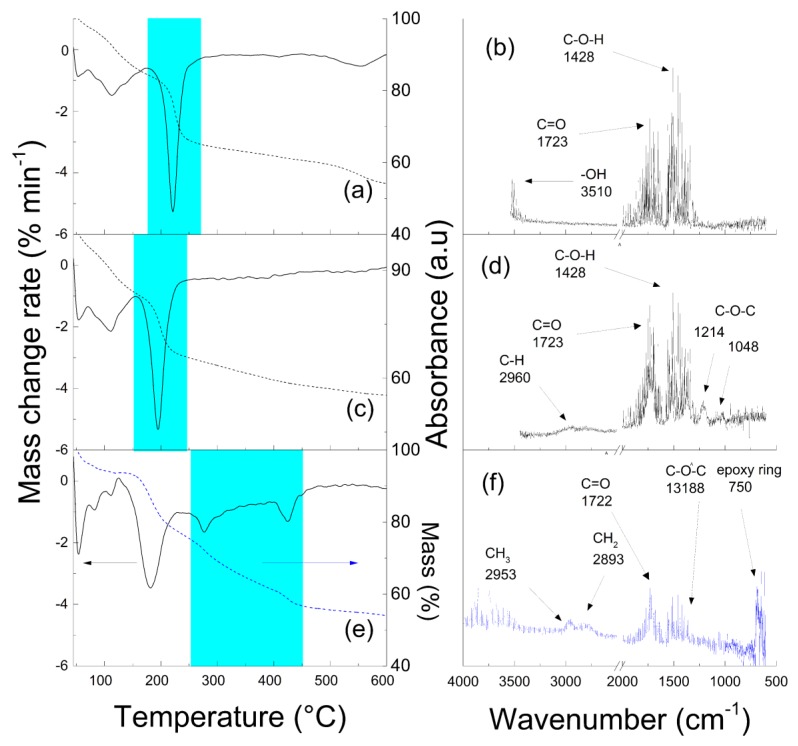
TGA analysis (**a**,**c**,**e**) with on-line monitoring of the FTIR spectra (**b**,**d**,**f**) for neat GO (**a**,**b**); GO-iniciator, and (**c**,**d**) and GO-PGMA (**e**,**f**) particles.

The TGA-FTIR technique utilizing the on-line monitoring of the FTIR spectra during thermal decomposition of the neat GO (Figure 3a,b), GO with bonded initiator (Figure 3c,d), and GO-PGMA (Figure 3e,f) particles was employed to further prove the successful coating of GO with PGMA chains. In this case, the TGA spectra of the left side of Figure 3 correspond to the FTIR spectra on the right side of Figure 3. For the neat GO particles and GO with an initiator, the detailed description was already published elsewhere [41]. However, the following description is novel for GO-PGMA particles. It can be clearly seen that the particles peak around 200 °C, which corresponds to oxygen-containing groups that decrease as side information to prove the partial reduction of the GO-PGMA particles. Moreover, the presence of the successful coating is remarkable from Figure 3e when, in the range from 250 to 450 °C, we observed two peaks both corresponding to the thermal decomposition of PGMA. A similar range of PGMA decomposition was also found in the case of modification of carbonyl iron [42] as well as in the case of GO-PGMA modified through SI-ATRP where the mass loss in Figure 3e is very similar to the following reference [27]. In case of GO-PGMA-2, the TGA-FTIR scan looked nearly identical because, from the structural point of view, the FTIR spectra in Figure 3f represent the collection of the signal during the period highlighted in Figure 3e by a cyan color. Specific absorption bands were found at 1722 cm^−1^ (carbonyl), 1318 cm^−1^ C-O-C stretching, 2961, and 2791 cm^−1^ (alkyl vibrations). Furthermore, the sharp peak at 750 cm^−1^ is based on an epoxy ring absorption and, therefore, confirms the presence of PGMA polymer chains grafted on the surface of GO.

**Figure 4:**
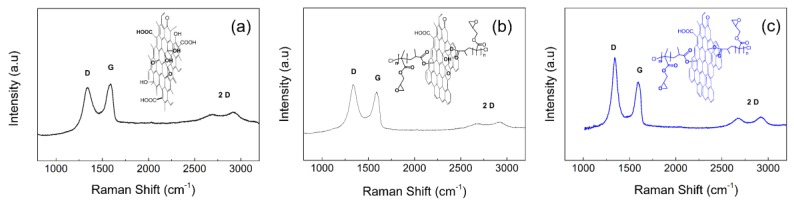
Raman spectra of the neat GO (**a**) GO-PGMA 1; (**b**) GO-PGMA 2; and (**c**) particles and corresponding chemical structures.

Graphene oxide reduction is a very important factor from its applicability point of view. If the material has a higher electric conductivity, the thermal conductivity is higher. The thermal energy redistribution in the sample is a crucial factor for the final photo-actuation performance. In addition, the composite material upon its deformation can show a sensing capability only if certain resistivity of the material is reached. Therefore, the high conductivity of the final composite is very important. This is similarly observed in the study by Georgousiss et al. [43]. Therefore, the conductivity and Raman spectroscopy (Figure 4) were investigated to prove the degree of reduction of GO and variously reduced GO-PGMA particles. The intensities ratios (I_D_/I_G_) were found to be 0.9 for neat GO (Figure 4a) and 1.08 and 1.26 for GO-PGMA 1 (Figure 4b) and GO-PGMA 2 (Figure 4c) corresponding to the determined conductivities of 1.2 × 10^−8^, 5 × 10^−7^, and 2.3 × 10^−3^ S·cm^−1^, respectively. The ratios indicate a significant and controllable reduction during the SI-ATRP process, which is very promising for further industrial applications.

**Figure 5:**
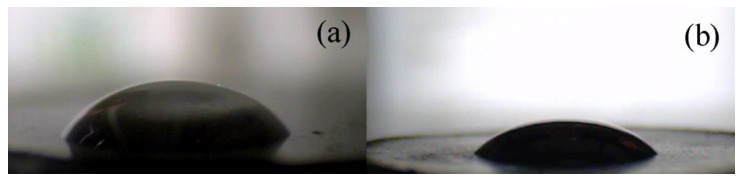
Images from CCD camera of the 5 µL PDMS droplets on the neat GO (**a**) and GO-PGMA (**b**).

Compatibility of the filler with a matrix is a crucial factor for improvement of the physical as well as mechanical properties of the final composite material. Inhomogeneity present in the sample due to the weak compatibility can be mainly responsible for mechanical instabilities during a repeatable mechanical load or, in the case of materials with desired electrical or thermal conductivities, can lead to their suffered performances. Therefore, the contact angle values of PDMS drops, as the elastomeric matrix used in this study, onto the surface of pellets prepared from the neat GO or GO-PGMA particles, which were investigated (Figure 5). It can be clearly seen that the contact angle of neat GO was determined to be 49.9° ± 3.2°, which showed relatively poor compatibility while the modification of the GO with short polymer chains of PGMA decreased the contact angle to 40.1° ± 1.3° and, therefore, improved the wettability between the PDMS and GO-PGMA surface due to the presence of the aliphatic polymer backbone. In this case, the influence of various conductivities was not proven and contact angles were in the range of error. However, the contacts angles were not so low as was previously found by our research group for PMMA or PBMA grafts [36].

**Figure 6:**
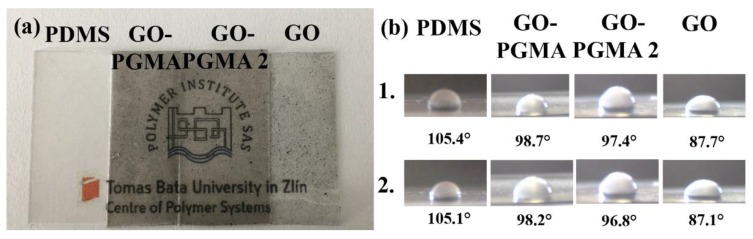
(**a**) Images of the neat PDMS and prepared composites and (**b**) Images from CCD camera of the 5 µL water droplets on the neat PDMS and various composites. Line 1 represents measurements for as-prepared samples while line 2 represents the measurements of the samples after 7 days of storage at RT.

In order to show how the optical properties of the composites were changed after adding various fillers, the images of neat PDMS as well as the GO-based composites were performed (Figure 6a). It can be seen that the best optical properties had a neat PDMS. However, the modification using the SI-ATRP approach provided the composites with better dispersed filler in comparison to the neat GO due to the fact that neat GO/PDMS composites seem to be of higher transparency with relatively big agglomerates and less uniform filler dispersion compared to the GO-PGMA grafted particles. In addition, the change of the contact angles between the water and composites is nearly negligible and is more affected by the modification than the time of storage (Figure 6b). This means that the properties that were changing over 7 days did so negligibly. The contact angle for neat as-prepared PDMS and after 7 days of storage is 105.4° and 105.1°, respectively. Similar results were found for other composites where the as-prepared GO-PGMA 1 after 7 days of storage show contact angles of 98.7° and 98.2°, respectively. The longer grafts provided composites with a slightly lower contact angle of 97.4° and 96.8° for as-prepared GO-PGMA 2 and after 7 days of storage, respectively, due to the higher amount of the hydrophilic epoxy groups presented on the GO-PGMA 2 particles surface. The lower contact angle was observed for neat GO, which was expected due to the presence of hydroxyl, carboxyl, and epoxy groups on its surface. Therefore, showing contact angle of 87.7° and 87.1° for as-prepared GO composite after 7 days of the storage, respectively.

**Figure 7:**
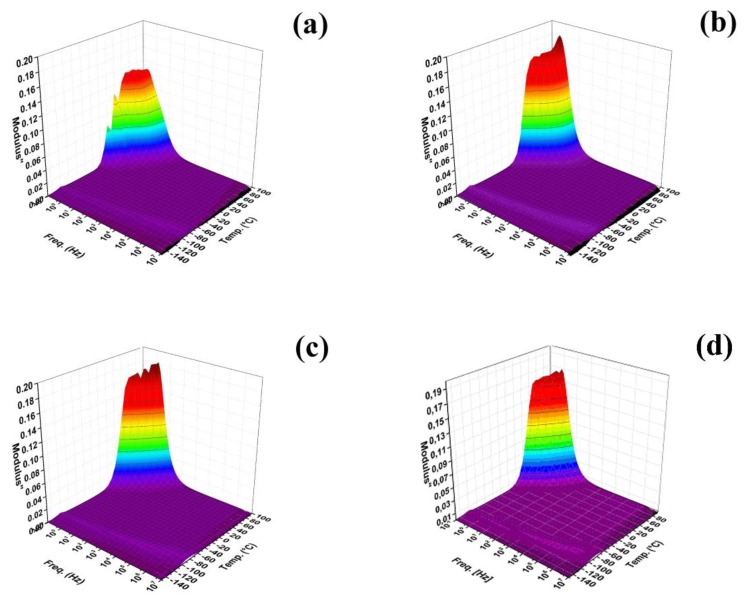
3D plots of the dielectric properties of the neat PDMS matrix, (**a**) neat GO-PDMS; (**b**) GO-PGMA 1; (**c**) and GO-PGMA 2; (**d**) All at 0.1 vol. % content in PDMS.

Investigations of dielectric properties (Figure 7) is very important due to the fact that, with the help of measurement in a broad temperature range and frequency-dependent permittivity, it is possible to calculate *E*_a_ of the glass transition and *T*_g_, which provides information about the polymer chain flexibility in the presence of GO particles. In our study, however, the prepared composites exhibited a rather strong electrical response to the applied electric field and, therefore, obtained *ε′* and *ε″* exhibited electrode polarization and were not suitable for *E*_a_ calculation [40]. Therefore, the expression of *M′* and *M″* was used, which was mainly *M″* according to Equation (2). Therefore, this quantity was also used for the interpretation of the dielectric spectra in Figure 5. Here the peak around −120 °C indicating the presence of *T*_g_ can be seen for all investigated samples.

From the position of the maxima of this peak at various temperatures, the *E*_a_ of *T*_g_ was calculated according to Equation (1) and results are listed in Table 1. It can be clearly seen that the highest *E*_a_ of 45.70 kJ·mol^−1^ was found for neat PDMS by following the neat GO composite with *E*_a_ of 36.57 kJ·mol^−1^. The sample GO-PGMA 1 with 0.1 volume percentage of particles of lower conductivity possess only a slight decrease in *E*_a_ showing an improved PDMS chain mobility after modification while the sample GO-PGMA 2 with same particle loading. However, with significantly higher conductivity possesses significantly better chain mobility and, lastly, the lowest *E*_a_ of 23.80 kJ·mol^−1^ further enhanced the photo-actuation capability. These results indicate that the presence of the GO-PGMA particles in the composite significantly improves the flexibility of the PDMS chains due to the fact that the energy necessary for the polymer chain movement is lower. As it will be shown below, these results were also confirmed by the investigation of DMA and photo-actuation performance since the results from these experiments well-correlates with these dielectric investigations.

**Figure 8:**
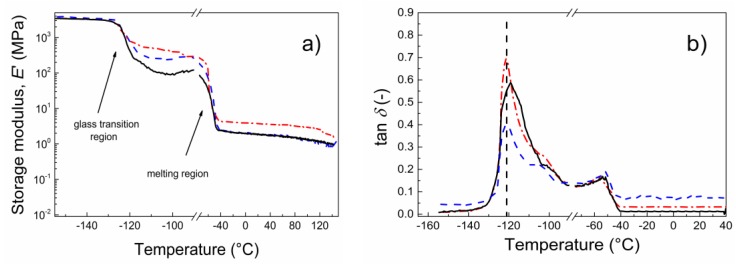
Dependence of the storage modulus (**a**) and tan δ (**b**) for broad temperature range for neat PDMS (black solid line) and for PDMS composites containing 0.1 vol % of neat GO (red dash dot line) and GO-PGMA 2 (blue dashed line).

Dynamic mechanical properties (Figure 8) are one of the most crucial factors influencing the applicability of materials as photo-actuation systems. Reversible contraction/elongation of the sample by light stimulation is a rather dynamic process and, therefore, investigation upon dynamic conditions is very important. As can be seen in Figure 8a, the storage moduli below *T*_g_ were nearly the same for all investigated samples. The first difference can be seen above *T*_g_ when the lowest storage modulus was obtained for neat PDMS and was followed by the GO-PGMA sample. The highest values were observed for samples containing neat GO particles. A similar situation occurs above the melting region when the neat GO possessed the highest storage modulus. This observation can be explained by possible covalent bonding between the PDMS and hydroxyl groups of GO particles [44]. Since the amount of the hydroxyl groups significantly decreased due to both the modification with PGMA chains and the reduction of the GO (clearly seen from Figure 3 and Figure 4), the mechanical response is mostly based on the good compatibility of the GO-PGMA particles with the PDMS. From the temperature dependence of the tan δ (Figure 8b), it can be seen that the peak of tan δ for the sample containing GO-PGMA decreased when compared with pure PDMS or PDMS filled with neat GO, which indicated enhanced compatibility of GO-PGMA with the PDMS matrix. Yet, the absolute value of tan *δ* after all transitions was higher for GO-PGMA than for PDMS matrix and PDMS containing neat GO due to the fact that physical entanglements of PGMA short polymer chains provide more flexible structure than in the case of GO with possible covalent bonding to the PDMS. Composites consisting of various GO-PGMA with different conductivities show nearly the same mechanical behavior. The differences were in the range of error. Therefore, only the one consisting of GO-PGMA particles with higher conductivities are shown in Figure 8. Therefore, it can be stated that GO-PGMA/PDMS composite provides a system with enhanced damping and also with improved flexibility of the composite system, which well-correlates with dielectric studies and which is in the same time highly suitable for the photo-actuation point of view.

**Figure 9:**
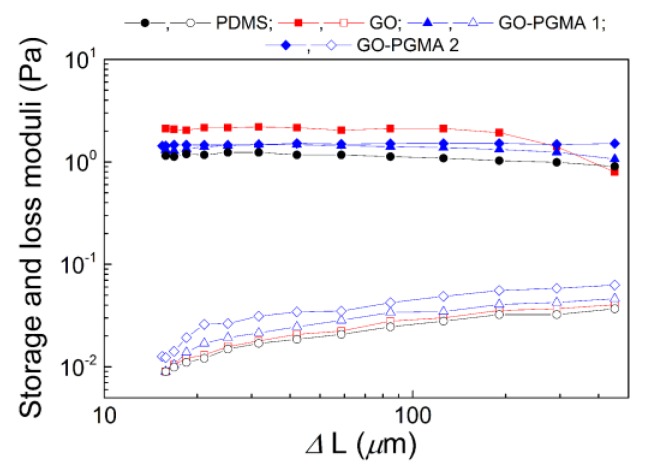
Dependence of the storage (solid symbols) and loss (open symbols) moduli on the change in deformation upon dynamic tensile testing at 1 Hz and 25 °C.

In order to investigate the mechanical properties of the prepared composites, dependence on the change in the length ∆*L* in the range of potential photo-actuation on the viscoelastic moduli were measured. Various natures of the filler can provide the system with multiple mechanical performances at the strain deformation obtained during light-stimulation. As can be seen in Figure 9, the composite consisting of the neat GO particles has the highest values of the storage modulus. However, the significant drop is visible at lower deformations. However, the GO-PGMA-based composites show lower values, which corresponds to their softer nature and higher possible elongation. According to the performed measurements, it can be expected that GO-PGMA composites will provide systems with better photo-actuation performance due to more suitable mechanical properties.

According to the previous study [45], the thermal conductivity is another crucial parameter affecting the photo-actuation performance. As can be seen from Table 2, the pure PDMS matrix has thermal conductivity of 0.071 W·m·K^−1^. It increases with the addition of filler for both investigated samples. Due to the improved compatibility of GO particles with the PDMS matrix by PGMA chains grafted on the GO surface, these composites with GO-PGMA exhibited further improvement of thermal conductivity compared with the composite based on neat GO (Table 2). Therefore, the composite sample containing GO-PGMA particles could provide significant contribution to the enhanced photo-actuation capabilities due to the improved heat redistribution within the sample.

**Figure 10:**
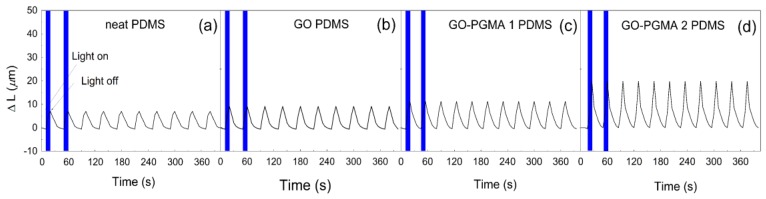
Photo-actuation performance of pure PDMS (**a**) and PDMS composites containing 0.1 vol.% of neat GO; (**b**) GO-PGMA-1; (**c**) and GO-PGMA-2 (**d**) at 6 mW irradiation intensity.

In order to confirm the photo-actuation ability of the GO-PGMA/PDMS composite, the photo-actuation performance in this case expressed as the change in the length ∆*L* was investigated (Figure 10). It can be seen that there was only a slight increase of the photo-actuation performance for PDMS containing neat GO compared with the neat PDMS provided that ∆*L* is 9.1 and 7.1 µm, respectively. The situation was considerably different in the case of the sample containing GO-PGMA particles (Figure 10c,d). The ∆*L* after irradiation reached a value of 20.2 µm, which is nearly three times higher than that obtained for the neat PDMS matrix (Figure 10a) and more than two times higher than that for PDMS composite with neat GO particles (Figure 10b). This significant enhancement is caused by several factors: (i) the particles has better wettability to PDMS and, therefore, better compatibility of the filler with the matrix, (ii) the presence of PGMA prevents the additional cross-links presented in the PDMS or GO/PDMS providing the system with more flexible structure, and (iii) increased electrical conductivity due to GO reduction during the SI-ATRP modification of GO leading to the enhanced thermal conductivity. The first and the third factors are responsible for more effective heat transport and homogeneous heat redistribution. The second mentioned factor ensures good flexibility, which is very important for reversible actuation and positively influences the overall performance.

**Figure 11:**
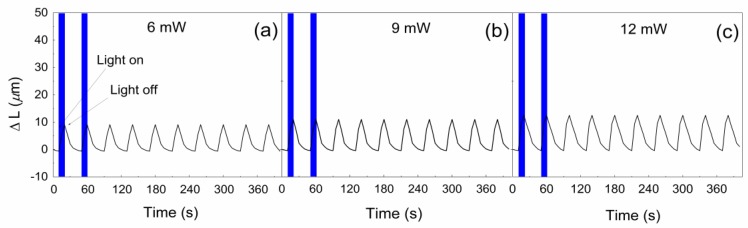
Photo-actuation performance of GO/PDMS composite at (**a**) 6 mW; (**b**) 9 mW; and (**c**) 12 mW irradiation intensity.

With increased intensity of the radiation, the difference between the investigated systems became even more pronounced. First, it should be mentioned that for neat PDMS, the changes were negligibly affected by the increasing intensity (results are not presented). The PDMS composite containing neat GO showed only a slight increase of ∆*L* in the line 9.1, 10.7, and 12.5 µm for 6, 9, and 12 mW, respectively (Figure 11).

**Figure 12:**
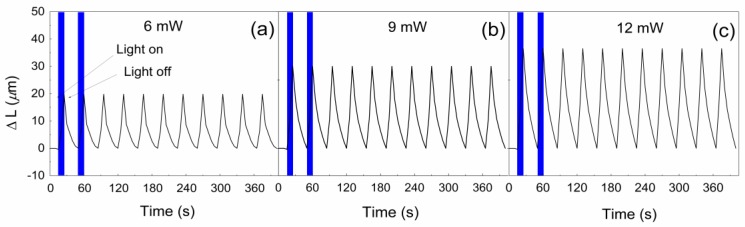
Photo-actuation performance of GO-PGMA 2/PDMS composite at (**a**) 6 mW; (**b**) 9 mW; and (**c**) 12 mW irradiation intensity.

However, the PDMS containing GO-PGMA showed significant enhancement in photo-actuation performance with ∆L in the line 20.2, 30.2, and 36.7 µm for 6, 9, and 12 mW, respectively (Figure 12). The main reason for this behavior is possible covalent interactions of the neat GO and PDMS already observed from DMA investigations. Therefore, it can be stated than the developed composite system based on the PDMS matrix containing GO nanoparticles simultaneously reduced and grafted with PGMA chains provided the reversible photo-actuation phenomenon and has excellent mechanical properties in a dynamic mode, which is highly promising for their potential applications.

## 4. Conclusions

In this paper, the successful grafting of the PGMA on the surface of the GO particles was performed by SI-ATRP and confirmed using TGA-FTIR and TEM investigations. Simultaneous reduction of GO during SI-ATRP was proven by Raman spectroscopy and electrical conductivity measurements. The enhanced compatibility of PDMS with GO after its modification with PGMA chains was confirmed by the contact angle measurements of PDMS drops on the GO and GO-PGMA pellets. Lower *E*_a_ of *T*_g_ of PDMS chains in the GO-PGMA/PDMS composite calculated from dielectric measurements compared with neat PDMS or GO/PDMS composite pointed to higher polymer chain dynamics thanks to the incorporation of GO-PGMA nanoparticles. Important mechanical performance upon dynamic conditions was investigated using DMA at a broad temperature range and it was found that GO surface modification provides the materials with enhanced damping properties, which positively affects the photo-actuation performance. Lastly, the photo-actuation capability measurements showed significantly improved performance of GO-PGMA-based composites due to the improved dispersibility, enhanced flexibility, and increased thermal conductivity. All these aspects provided the system with very promising performance and with values suitable for real-life applications.

## Figures and Tables

**Table 1:** Activation energies of a glass transition process for pure PDMS and PDMS composites with variously conducting particles.

Sample Code	*E*_a_ (kJ·mol^−^^1^) of *T*_g_ (−120 °C)
pure PDMS	45.70
0.1 vol % neat GO/PDMS	36.57
0.1 vol % GO-PGMA 1/PDMS	31.32
0.1 vol % GO-PGMA 2/PDMS	23.80

**Table 2:** Thermal conductivity of pure PDMS and PDMS composites with various fillers.

Sample Code	Thermal Conductivity (W·m·K^−1^)
pure PDMS	0.071
0.1 vol % neat GO/PDMS	0.144
0.1 vol % GO-PGMA-1/PDMS	0.152
0.1 vol % GO-PGMA-2/PDMS	0.161
